# A comprehensive dataset of *Halyomorpha halys* for automated detection and monitoring in orchards

**DOI:** 10.1038/s41597-026-07774-3

**Published:** 2026-07-15

**Authors:** Lorenzo Palazzetti, Francesco Betti Sorbelli, Papiya Das, Lennart Almstedt, David Niederprüm, Lars Wolf, Cristina M. Pinotti

**Affiliations:** 1 Pennsylvania State University, University Park, USA; 2 University of Perugia, Perugia, Italy; 3 University of Florence, Florence, Italy; 4 Technische Universität Braunschweig, Braunschweig, Germany

## Abstract

The *Halyomorpha halys* (HH) (Brown marmorated stink bug (BMSB)) is a globally invasive pest responsible for severe economic losses in fruit production, yet open, large-scale monitoring datasets remain scarce. Here we present a multi-year, multi-modal dataset acquired over three consecutive growing seasons in an experimental pear orchard in Carpi (Modena), Italy, designed to support research in entomology, agriculture, and Machine Learning (ML). The release comprises (i) three years of Unmanned Aerial Vehicle (UAV)-acquired RGB imagery annotated with bounding boxes for HH; (ii) one season of stationary canopy-level imaging from five fixed cameras targeting insects in occluded regions; (iii) temporally aligned environmental time series of microclimatic variables; and (iv) complementary 2021 acquisitions including smartphone field images, laboratory images, and a synthetic subset generated by compositing insect silhouettes onto orchard backgrounds. The second and third years follow a standardized UAV protocol, while the first year combines heterogeneous consumer-grade modalities. All annotations were manually verified, and the public release includes only images containing at least one annotated insect. This multi-modal design supports applications including small-object detection, sensor fusion, behavioral analysis, and data-driven pest-management tools.

## Background & Summary

The *Halyomorpha halys* (HH) is an invasive agricultural pest of global significance^1^. Since its introduction into Europe, HH has rapidly expanded its distribution, causing substantial economic losses to fruit crops, particularly pears, apples, and stone fruits^2^. The species’ high mobility, broad host range, and tendency to remain concealed within complex canopies make it challenging to detect and monitor using conventional field scouting methods^3^. Accurate, scalable, and non-intrusive monitoring tools are therefore essential for understanding HH population dynamics and for supporting integrated pest-management strategies.

Despite the ecological and economic importance of HH, the availability of open, large-scale datasets for automated monitoring remains very limited. To our knowledge, only two publicly available datasets specifically targeting HH exist at the time of writing, both hosted on community-driven annotation platforms^4,5^. These collections share several critical limitations that constrain their applicability in rigorous research. Images were acquired exclusively with consumer high-end cameras and without a standardized imaging protocol, resulting in heterogeneous viewpoints, distances, and illumination conditions. The number of annotated samples is small, limiting the statistical representativeness of each collection. Most importantly, the images predominantly depict HH at very close range, where the insect occupies a large fraction of the image frame. This acquisition style is fundamentally at odds with real-world field monitoring conditions, where the pest appears as a small, often partially occluded object against complex canopy backgrounds and must be detected reliably at scale across large orchard areas. Existing entomological datasets more broadly are often restricted in scale, rely on a single sensing modality, or lack standardized and verified annotations, limiting reproducibility, benchmarking, and deployment. No publicly available resource combines multi-year, multi-sensor HH observations collected under realistic field conditions with temporally aligned environmental measurements, leaving a substantial gap for ecological modeling, longitudinal analysis, and benchmarking of vision-based monitoring systems.

Recognizing the need for scalable, automated, and reliable monitoring systems, the ICT-AGRI-FOOD ERA-NET Co-fund Haly.ID project^6^ was launched in 2021 with the objective of reducing growers’ dependence on traditional traps and manual sampling by 2024. The project emphasizes the use of Internet of Things (IoT), Unmanned Aerial Vehicles (UAVs), embedded vision systems, and Machine Learning (ML) algorithms to support real-time, data-driven decision-making in orchards^7^.

As an outcome of this project, we present a longitudinal, multi-modal collection of HH monitoring data acquired over three consecutive growing seasons in an experimental pear orchard. The dataset integrates aerial RGB imagery, stationary canopy-level cameras, environmental sensor data, and curated annotations, forming a unique resource for ecological, agricultural, and ML research. To support early-stage model development and methodological exploration, the release also includes a synthetic image subset generated by compositing insect silhouettes onto orchard background scenes.

Capturing HH within tree canopies presents unique challenges: insects are small, cryptic, and often positioned on the underside of leaves or within shaded regions. We therefore adopted UAV-based RGB imaging to obtain rapid, repeated, and scalable coverage of the orchard canopy without disturbing insect behavior, while maintaining compatibility with widely used computer-vision pipelines. In parallel, stationary cameras were deployed to monitor insects in occluded or low-visibility regions of the canopy, providing complementary close-range observations that aerial platforms cannot consistently capture. Across the three-year period, imaging protocols evolved in response to operational constraints and system maturation, enabling longitudinal analysis under both heterogeneous and standardized acquisition conditions. By combining aerial, stationary, and environmental sensing, the dataset supports applications ranging from large-scale field mapping to fine-grained behavioral analysis.

## Methods

In this section, we describe the procedures used to generate and acquire all components of the dataset, including the characteristics of the field site, the protocols adopted for image collection using smartphones, UAVs, and stationary cameras, and the deployment of the environmental monitoring system. This study did not involve human participants, patients, or personal data; therefore, human-data consent procedures were not applicable.

### Field Site

Data were collected over a three-year period (2021–2023) in Carpi, Modena, Italy, within an experimental pear orchard (approximately 44.729936° N, 10.874855° E) covering approximately 1700 m^2^. The monitored orchard in this release corresponds to a single pear cultivar. The orchard was maintained under conditions that facilitated continuous monitoring of HH activity. No insecticide treatments were applied during the study period, and pheromone lures were deployed in accordance with standard integrated pest-management guidelines.

The orchard consisted of 12 rows of 15 pear trees each (approximately 180 trees). Rows were spaced 4 m apart and trees within each row were spaced 2.5 m apart. This stable spatial layout enabled repeated and comparable monitoring across seasons. Climatic conditions typical of the Emilia–Romagna region, including warm summers, a humid continental climate, and seasonally varying canopy density, were naturally preserved. A regional flooding event affected soil moisture and microclimatic conditions during part of the 2023 season but did not interrupt data collection. To provide site-specific climatic context, we analyzed public observations from the ARPAE-SIMC station *Cortile di Carpi* (reference point for the experimental area) for 2021–2023. At annual scale, total precipitation ranged from 441.8 to 617.2 mm, mean air temperature from 13.14 to 15.04 °C, annual minimum temperature from  − 8.9 to  −6.6 °C, annual maximum temperature from 37.6 to 38.0 °C, and mean relative humidity from 72.37% to 73.44%. Table 1 reports monthly climatological summaries across 2021–2023 (mean, standard deviation, minimum, and maximum across years).

**Table 1:** Monthly climate characterization for the Cortile di Carpi reference station (2021–2023).

Month	Air temperature mean (°C)	Relative humidity mean (%)	Precipitation total (mm)
Jan	2.93 ± 1.85 [1.81, 5.07]	84.89 ± 0.30 [84.60, 85.20]	48.73 ± 25.00 [20.80, 69.00]
Feb	5.40 ± 0.81 [4.58, 6.19]	74.90 ± 2.79 [72.13, 77.72]	13.53 ± 3.43 [10.40, 17.20]
Mar	8.28 ± 1.59 [6.99, 10.05]	66.20 ± 4.11 [63.05, 70.85]	17.27 ± 15.14 [2.80, 33.00]
Apr	11.51 ± 0.53 [10.90, 11.88]	70.96 ± 1.15 [69.64, 71.63]	43.00 ± 23.52 [17.00, 62.80]
May	17.68 ± 1.56 [16.50, 19.45]	73.49 ± 5.43 [68.71, 79.39]	109.53 ± 77.17 [59.40, 198.40]
Jun	23.44 ± 0.97 [22.46, 24.40]	66.65 ± 3.78 [63.57, 70.87]	32.07 ± 19.23 [16.60, 53.60]
Jul	25.55 ± 0.96 [24.58, 26.49]	64.61 ± 6.45 [57.18, 68.71]	30.93 ± 1.72 [29.40, 32.80]
Aug	24.11 ± 0.63 [23.39, 24.54]	65.98 ± 1.68 [64.04, 66.97]	79.33 ± 66.15 [8.80, 140.00]
Sep	19.78 ± 0.95 [18.90, 20.79]	73.69 ± 2.28 [72.18, 76.32]	31.00 ± 21.33 [7.00, 47.80]
Oct	15.70 ± 3.40 [12.03, 18.74]	78.93 ± 5.47 [72.70, 82.97]	8.47 ± 11.56 [1.20, 21.80]
Nov	9.63 ± 1.70 [8.43, 11.57]	82.52 ± 10.50 [70.45, 89.46]	62.07 ± 53.32 [0.80, 98.00]
Dec	4.46 ± 1.64 [2.60, 5.72]	87.98 ± 4.32 [83.77, 92.41]	53.33 ± 45.94 [9.60, 101.20]

Values are reported as mean  ± standard deviation [minimum, maximum] across years.

### Data Acquisition

**Fig. 1:**
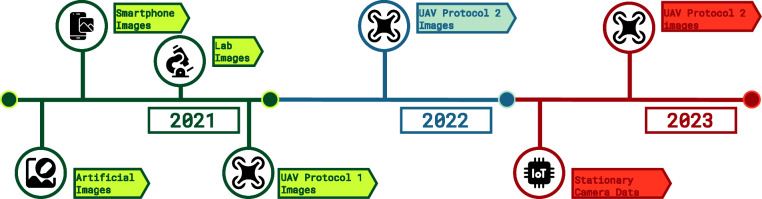
Acquisition timeline for the full dataset.

Figure 1 summarizes the different subsets that collectively compose the full dataset and their temporal coverage.

#### Artificial Data

At the beginning of the project (2021), no aerial imagery was available for training object-detection models. To support early-stage model development, we constructed a synthetic dataset by compositing insect silhouettes onto orchard background images sourced from Pexels; only the resulting synthetic composites are redistributed as part of this dataset, while original third-party background files are not redistributed. This design is consistent with the applicable stock-content licensing model (use and modification allowed, standalone redistribution restricted). Silhouettes were extracted and algorithmically placed using a Python script that randomized position, scale, and the number of insects per image. A total of 221 background images and 105 silhouettes were used, resulting in 8, 880 synthetic images^8^. This subset is intended to support algorithmic development and is not used for ecological or longitudinal analyses.

#### Smartphone and Laboratory Data

To complement the synthetic data during the first year, team members captured images of HH specimens in the orchard using smartphones and professional cameras, resulting in 299 field images. After quality control and exclusion of empty-label smartphone samples, 273 smartphone images are retained in the released annotated subset. An additional 80 images were acquired under laboratory conditions using live or preserved specimens. In total, 353 real annotated images from these two modalities are included in the release.

#### UAV-based Data

**Fig. 2:**
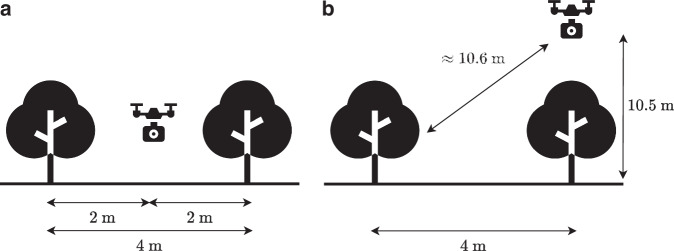
Illustration of the UAV acquisition protocols.
- *2022–2023 protocol (Protocol 2)*. Starting in 2022, an overhead imaging strategy replaced the lateral protocol due to operational constraints imposed by the collision-avoidance system^12,13^. The UAV followed an autonomous flight plan at 10.5 m above the canopy and paused at predefined waypoints (Fig. 2). At each waypoint, 20 images of the corresponding tree crown were captured. The 2022 and 2023 campaigns produced 402 and 164 images, respectively.

UAV acquisitions were performed using a DJI Matrice 300 RTK UAV^9^ equipped with a DJI Zenmuse H20 camera^10^. Flights were conducted during the peak HH activity period in each year. Missions were generally executed under favorable weather (no rain and limited wind), and typically around high-sun daytime windows to reduce low-light effects and strong directional shadows across orchard rows. During periods of unfavorable weather, flights were postponed. Two acquisition protocols were adopted:

- *2021 protocol (Protocol 1)*. During the first year, the UAV was manually piloted along each tree row^11^. One image per tree was captured at a fixed stand-off distance and an altitude of 1.5 m using a manual optical zoom setting (Fig. 2). This protocol produced 855 images in total; after excluding empty-label samples, 653 annotated images are retained in the released UAV-2021 subset.

Although a larger number of images were acquired during UAV operations, only frames containing at least one annotated insect were included in the public release.

#### Stationary Camera Data

In 2023, five stationary canopy-level cameras were deployed within the orchard and remained fixed throughout the observation period. Each unit consisted of a Raspberry Pi (RPi) single-board computer connected to a fixed-focal-length camera module^14^. The devices were positioned to image fruit-bearing canopy regions, including partially occluded areas. Images were captured at regular intervals throughout the day, with several frames per hour during the main activity period of HH, and transmitted to a local gateway for storage.

#### Microclimate Weather Data

Micro-environmental monitoring was conducted in 2023 using a small network of RPi-based sensor nodes deployed within the orchard and installed in proximity to the stationary cameras. The network comprised five sensor nodes distributed across the field, with multiple sensor boards mounted at different heights within the canopy region. Sensors measured air temperature, relative humidity, barometric pressure, light intensity, and leaf wetness. Sensor boards were installed at heights ranging approximately from 0.7 m to 2.0 m above ground level and housed in radiation-shielded enclosures. Measurements were acquired at short intervals and transmitted via MQTT to a central gateway. The resulting time series is temporally aligned with the stationary camera imagery.

## Data Records

The dataset is publicly available through the Zenodo repository^15^. The concept DOI is 10.5281/zenodo.18045392, while the version associated with this revised release is 10.5281/zenodo.20431348.

The dataset is distributed as eight core compressed archives. The directory structure is as follows:

- artificial.zip
- laboratory.zip
- smartphone.zip
- stationary.zip
- uav-2021.zip
- uav-2022.zip
- uav-2023.zip
- weather-data.zip

In addition to the eight core archives above, the current Zenodo version also includes four representative negative-image archives: negative-uav-2021.zip (202 images), negative-uav-2022.zip (413 images), negative-uav-2023.zip (413 images), and negative-stationary.zip (1,740 images). These negative archives are image-only subsets and do not include annotation files. A complete release of all insect-free frames is not currently feasible in Zenodo due to record-size limits (50 GB), while the full negative pool exceeds 200 GB; therefore, the released negatives are representative subsets with explicit sampling criteria. Annotated (positive) frames represent only a small fraction of all acquired frames: for the two most relevant stationary cameras they account for approximately 0.8% and 0.6% of the acquired frames, and UAV acquisitions fall within the same range. The repository also includes official predefined train/validation/test split files, generated with a fixed seed and a 70/20/10 partition:

- artificial_split.json
- smartphone_split.json
- laboratory_split.json
- stationary_split.json
- uav-2021_split.json
- uav-2022_split.json
- uav-2023_split.json
- split_summary.json
- image_metadata.csv
- weather_sample_2023-06-01.csv
- weather_field_schema.csv
- weather_restore_and_query.md
- weather_compose.yaml

For uav-2021 and smartphone, split generation is stratified by class presence (HH/*Nezara viridula* (NZ)) on the filtered non-empty annotated pools. A root-level file named image_metadata.csv is also provided, containing one record per image with archive name, relative path, filename, modality, year, synthetic/real indicator, negative-subset indicator, annotation availability, and third-party provenance/license fields.

All ZIP files, with the exception of weather-data.zip, contain image data alongside their corresponding annotation files, except for the four negative-image archives described above. As each archive contains a single top-level directory, we will hereafter refer to them simply as **directories**.

The weather-data/ directory contains environmental measurements recorded by a network of five stationary sensor nodes deployed within the orchard, while the influx-backup/ subdirectory stores archived time-series records exported from the InfluxDB database.

Image data across the artificial/, laboratory/, smartphone/, stationary/, and uav-2021/, uav-2022/, and uav-2023/ directories are stored in JPEG (.jpg) and PNG (.png) formats. Annotations are provided in plain-text (.txt) files following the You Only Look Once (YOLO) annotation format. For each image file, a corresponding annotation file with the same base filename is provided. Images and annotation files are organized into images and labels subdirectories, respectively.

The annotation files contain object-level bounding box annotations for two insect species in the adult stage: HH and NZ. Class identifiers are encoded as integer labels, where class 0 corresponds to HH and class 1 corresponds to NZ. Annotations are defined on a per-image basis.

The core modality archives include images containing at least one annotated insect. Representative insect-free (negative) images are released separately in the four negative-image archives listed above.

Image filenames vary by acquisition setting and follow the conventions described below.

Images in the artificial/ directory are named using a simple numeric counter ranging from 0 to 8879. Each image is associated with a YOLO annotation file sharing the same numeric identifier.

Images acquired using UAVs and stored in the uav-2021/, uav-2022/, and uav-2023/ directories follow the naming convention:

DJI_<datetime>_<counter>_Z,

where <datetime> indicates the acquisition timestamp, <counter> is a sequential identifier, and Z denotes optical zoom imagery.

Images captured by stationary cameras are named according to the pattern:

<date>T<time>_<MAC-address>,

where <date> and <time> indicate the acquisition timestamp and <MAC-address> identifies the camera board.

Laboratory images follow the naming convention:

<background-type>_HH_<counter>,

where <background-type> denotes the background category used during acquisition, HH indicates *Halyomorpha halys*, and <counter> is a sequential index.

Images acquired using smartphones do not follow a standardized naming convention; however, each image can be uniquely matched to its corresponding annotation file through identical filenames.

Environmental microclimate data were collected by the stationary sensor network deployed within the orchard and archived in the directory weather-data/influx-backup/. The data are stored as .tsm files generated by the InfluxDB time-series storage engine and represent archived time-series measurements recorded by the nodes. To improve accessibility for non-database users, we additionally provide weather_sample_2023-06-01.csv (tabular sample extract), weather_field_schema.csv (field/unit description), weather_restore_and_query.md (reproducible restore/query/export workflow), and weather_compose.yaml (portable Docker Compose setup). In this setup, users define the environment variable WEATHER_DATA_DIR as the absolute local path to the weather-data directory before starting the containerized restore/query workflow.

Because a full static CSV/Parquet export of the environmental records is not feasible within Zenodo’s record-size limits, we instead release the complete time-series database together with a reproducible query workflow. By running the provided Docker Compose file (weather_compose.yaml), users restart a local InfluxDB service with the archived bucket already mounted, gaining full query access to the entire database. Listing 1 reports, for every sensor, all available attributes (host, sensor, and measured field),while Listing 2 extracts a specific measurement as a tabular stream. Both examples use the InfluxDB Flux query language; from this starting point users can compose arbitrary queries following the official documentation^1^.^1^

**Listing: 1** Listing all sensor attributes (host, sensor, field) available in the database.

**Listing: 2** Extracting a specific measurement (air temperature from sensor sht3x on node fritz).

Based on project technical documentation, the network comprised five Raspberry-Pi-based nodes, with sensing elements installed at canopy level (approximately 0.7–2.0 m above ground). Documented sensor components include SHT3x (air temperature and relative humidity), SI1141 (light intensity), and BMP384 (temperature and barometric pressure on Pi-HAT modules), together with an Eltako WS wind sensor and a low-cost resistive leaf-wetness probe (YL-83/MH-RD type, LM393-based) deployed on a subset of stations. Part of the sensor-board communication used I2C links, with a bridge to one-wire for longer-distance node wiring. These are commercially available sensing components commonly used in agro-environmental monitoring. Detailed per-node calibration states and complete maintenance logs are not consistently available in the archived files.

Table 2 summarizes the environmental variables available in the dataset together with their measurement units.

**Table 2:** Environmental variables included in the sensor-node microclimate dataset and associated sensing components.

Variable	Unit	Typical sensor family
Air temperature	°C	SHT3x, BMP384
Relative humidity	%	SHT3x
Light intensity	lx	SI1141
Barometric pressure	hPa	BMP384
Wind-related signal (anemometer)	sensor units	Eltako WS
Leaf wetness	%	YL-83/MH-RD

No additional auxiliary metadata files (e.g., UAV flight logs, GPS trajectories, or sensor calibration records) are included in the repository beyond the information embedded in the data files themselves; however, image_metadata.csv provides image-level provenance and modality metadata for all released image archives. Class label definitions are documented implicitly through the annotation format, where numerical class identifiers correspond to insect species as described above.

All data files are provided in their original recorded formats, without filtering or post-processing beyond annotation generation.

## Data Overview

This section summarizes the composition of the dataset, including the number of images, annotations, and environmental records, and their distribution across acquisition modalities and time.

The dataset comprises three primary components:

- **Multi-year RGB imagery**. The collection spans three consecutive growing seasons and integrates multiple acquisition modalities. In 2021, the dataset includes synthetic images, smartphone acquisitions, laboratory captures, and manually piloted UAV flights. In 2022, images were collected exclusively through an automated UAV imaging pipeline. In 2023, the acquisition protocol expanded to include additional automated UAV flights. All UAV-derived images across the three seasons are fully annotated for HH, while NZ, a native Italian stink bug, is annotated only where present (observed in 2021).
- **Stationary canopy-level imaging**. One season (2023) of time-lapse imaging from five fixed cameras placed within the tree canopy. These image streams capture insects in highly occluded leaf regions, and all released frames are manually annotated for HH.
- **Environmental data**. One season (2023) of meteorological variables recorded by a small network of stationary sensor nodes in parallel with the stationary imaging. These time series provide a temporally synchronized record of environmental conditions and daily HH activity throughout the season of image acquisitions.

It is designed to support a broad range of applications, including ecological modeling, phenology prediction, sensor fusion, precision-agriculture decision support, and benchmarking of computer vision algorithms for small-object detection in complex outdoor environments.

Table 3 summarizes the number of images and annotated insects across the different acquisition settings included in the dataset. Bounding box (shortly, bb) sizes and positions are reported in normalized coordinates, and image dimensions are given in pixels.

**Table 3:** Summary of dataset composition by acquisition source and year.

Year	Set	# Images	# HH	# NZ	Avg. image size (W × H)	Avg. bb size (W × H)	Avg. bb position (x × y)
2021	Artificial	8, 880	79, 988	0	2001 × 1396	0.03 × 0.04	0.50 × 0.27
	Laboratory	80	320	0	3708 × 3246	0.07 × 0.08	0.49 × 0.50
	Smartphone	273	639	8	3053 × 3819	0.06 × 0.06	0.47 × 0.51
	UAV (Protocol 1)	653	1, 585	281	5184 × 3888	0.01 × 0.02	0.49 × 0.48
2022	UAV (Protocol 2)	402	661	0	5184 × 3888	0.01 × 0.02	0.48 × 0.51
2023	UAV (Protocol 2)	164	252	0	5184 × 3888	0.01 × 0.02	0.50 × 0.51
	Stationary	1, 681	3, 755	0	4056 × 3040	0.03 × 0.05	0.43 × 0.40

Average image size is rounded to the nearest pixel.

The artificial subset accounts for 8,880 of the 12,133 total images (approximately 72%) and for 79,988 of the 87,489 annotated insects (approximately 91%). This numerical dominance reflects the original motivation of the subset: at the start of the project, no real-world aerial imagery was available, and the synthetic data were generated as a stopgap to enable early-stage model development before field acquisitions began. The artificial subset should therefore be treated as an independent modality and is not suitable for ecological or longitudinal analyses. For computer vision purposes, it can be used in isolation to bootstrap training, or a portion of it may be combined with real-world subsets to augment the training pool and improve generalization; however, models should always be evaluated exclusively on real-world subsets. Indeed, as shown in^8^, the models trained on synthetic images only tend to fail in predicting on real-world data. Laboratory and smartphone acquisitions contribute comparatively fewer images, while UAV and stationary-camera data introduce real-world variability across outdoor conditions.

Comparing years, UAV imagery from 2021 (Protocol 1) includes both HH and NZ, whereas UAV data from 2022 and 2023 (Protocol 2) contain only HH. Image resolution is highest for UAV and laboratory images and lowest for artificial images, reflecting differences in acquisition devices and setups. Stationary cameras provide continuous coverage across multiple days, in contrast to the discrete UAV flight campaigns.

Overall, the dataset combines large-scale synthetic data, controlled laboratory images, heterogeneous smartphone acquisitions, continuous stationary-camera monitoring, and UAV imagery with substantial temporal and spatial variation. This diversity makes it well suited for training and evaluating insect detection models under a wide range of controlled and real-world conditions.

**Fig. 3:**
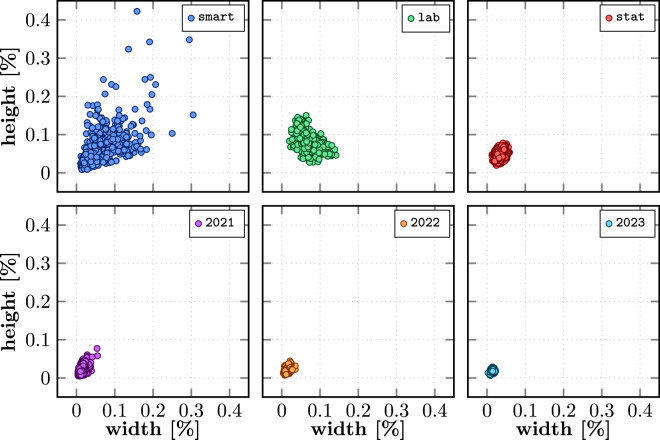
Distribution of bounding box sizes across acquisition settings.

To characterize the dataset at a high level, we analyze both geometric properties (e.g., bounding box dimensions) and image appearance features (e.g., blurriness and perceived brightness). Figure 3 summarizes the distribution of annotated insect bounding box sizes across the different acquisition settings. Bounding box width and height are reported along the *x*- and *y*-axes, respectively, and are expressed as percentages of the corresponding image dimensions.

Across all subsets, bounding box sizes are largely confined within 20% of the image width and height, indicating a broadly consistent scale of annotated objects. Despite this overall uniformity, distinct patterns emerge between acquisition modalities. Smartphone images exhibit the widest variability in bounding box dimensions, with a non-negligible number of instances exceeding 20% and reaching values of approximately 40%. As a result, the smart subset contains both the largest and the least standardized bounding boxes in the dataset, reflecting heterogeneous acquisition conditions and device-dependent variability.

Laboratory images also tend to feature comparatively larger bounding boxes; however, their size distribution is notably more constrained than that observed for smartphone images. This reduced variability is attributable to the controlled acquisition setup and the use of a single professional camera, which results in more consistent framing and object scale.

In contrast, in-field acquisitions display a highly standardized bounding box size distribution. The stationary camera subset (stat) contains bounding boxes consistently below 10% of the image dimensions, with a pronounced concentration around 5%. This clustering reflects the fixed camera placement and carefully calibrated deployment geometry, which ensure stable object scale despite environmental variability.

UAV imagery shows the highest level of standardization overall. While the 2021 UAV data (Protocol 1) exhibit moderate variability in bounding box sizes, the 2022 and 2023 collections (Protocol 2) display tightly clustered distributions, with most bounding boxes having nearly identical dimensions. This difference is consistent with the acquisition protocols: Protocol 1 involved lower flight altitudes, resulting in larger bounding boxes, whereas Protocol 2 employed higher and more uniform flight altitudes, yielding smaller and more consistent object sizes.

**Fig. 4:**
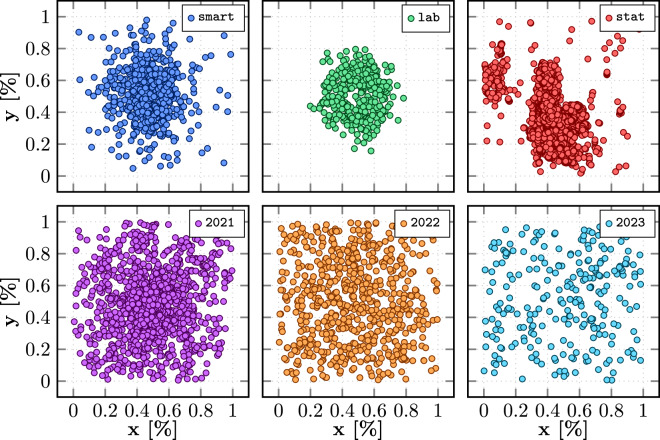
Spatial distribution of insect locations within image frames.

Figure 4 illustrates the spatial distribution of insect locations within the image frame. For each annotated instance, the *x*- and *y*-coordinates of the bounding box center are reported on the horizontal and vertical axes, respectively, and are expressed as percentages of the corresponding image dimensions.

In contrast to bounding box dimensions, bounding box positions exhibit substantial variability across acquisition settings. The greatest spatial dispersion is observed in UAV imagery, where bounding box centers span nearly the entire image plane. This pattern reflects the unconstrained spatial occurrence of HH in natural field conditions, where insect locations are not influenced by camera framing.

Stationary camera imagery displays a more structured spatial pattern, with bounding box centers predominantly concentrated in the lower central region of the image. This distribution is attributable to the fixed camera deployment, which consistently frames pear trees within this region of the scene. As pear fruits act as attractors for HH, insect instances are more frequently observed in these areas.

Manual acquisition modalities, including smartphone (smart) and laboratory (lab) images, show a markedly different behavior. In these subsets, bounding box centers are strongly clustered around the image center, with limited spatial dispersion. This reflects the inherent bias of manual image capture, where operators tend to center the subject of interest within the camera field of view rather than sampling locations uniformly.

**Fig. 5:**
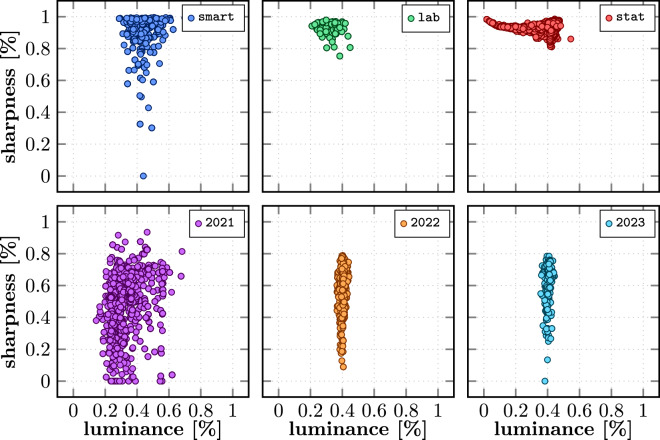
Image quality metrics across acquisition settings.

Figure 5 summarizes image quality metrics across acquisition settings, highlighting differences attributable to illumination conditions, sensor characteristics, and imaging geometry. To quantify basic image appearance properties, we compute normalized measures of luminance and sharpness from grayscale images. The *luminance* is defined as the mean grayscale intensity normalized to the unit interval, providing a resolution-independent estimate of overall image brightness, where values close to 0 indicate dark images, values close to 1 indicate bright images, and intermediate values correspond to balanced illumination. Image *sharpness*, used here as a proxy for blurriness, is measured using the variance of the Laplacian operator, which captures the presence of high-frequency content associated with edges and fine structural details. Sharpness values close to 0 indicate blurred images, while higher values correspond to sharper imagery.

Manually acquired images (smartphone and laboratory subsets) exhibit the highest luminance and sharpness scores, with most samples clustered around intermediate luminance values and near-maximal sharpness. This reflects the constrained nature of manual acquisition, in which camera settings and framing are adjusted by the operator to optimally capture the insect of interest.

Stationary camera imagery also shows consistently high sharpness scores, but with a wider spread in luminance values, including some underexposed images. This effect is primarily associated with early-morning acquisitions, when ambient light levels are lower. Despite reduced illumination in these cases, insect instances remain visually discernible and are therefore retained in the dataset.

UAV imagery displays well-balanced luminance distributions across all acquisition years, with minor variations observed in the 2021 data (Protocol 1), likely due to heterogeneous shading caused by canopy structure. In terms of sharpness, UAV images exhibit lower average scores compared to manually acquired data. This behavior reflects the global nature of the sharpness metric, which is sensitive to motion-induced artifacts, background texture, and out-of-focus regions unrelated to the annotated insects (e.g., branches close to the camera or transient platform motion). Visual inspection confirms that annotated insect instances are well-resolved, and images are retained accordingly. As both luminance and sharpness scores represent global image statistics, they capture overall imaging conditions but do not encode local quality variations at the object level, which are the primary focus of this dataset.

Overall, the dataset comprises a total of 12,133 images and 87,489 annotated insects, including 87,200 instances of HH and 289 instances of NZ. Stationary-camera imagery spans approximately 126 days of observation. Specifically, the first record is dated 2023-05-31, while the last is 2023-10-03. Within this period, stationary observations include a late-May/early-June phase with fully green canopies and very small pears (tail of the first HH generation), and a late-August/mid-September phase with mature pears and high HH abundance (second generation peak).

Environmental measurements were recorded at short intervals; however, due to sensor outages and transmission interruptions, some variables exhibit irregular sampling and discontinuous timestamps. Discontinuous intervals are preserved without gap filling.

**Fig. 6:**
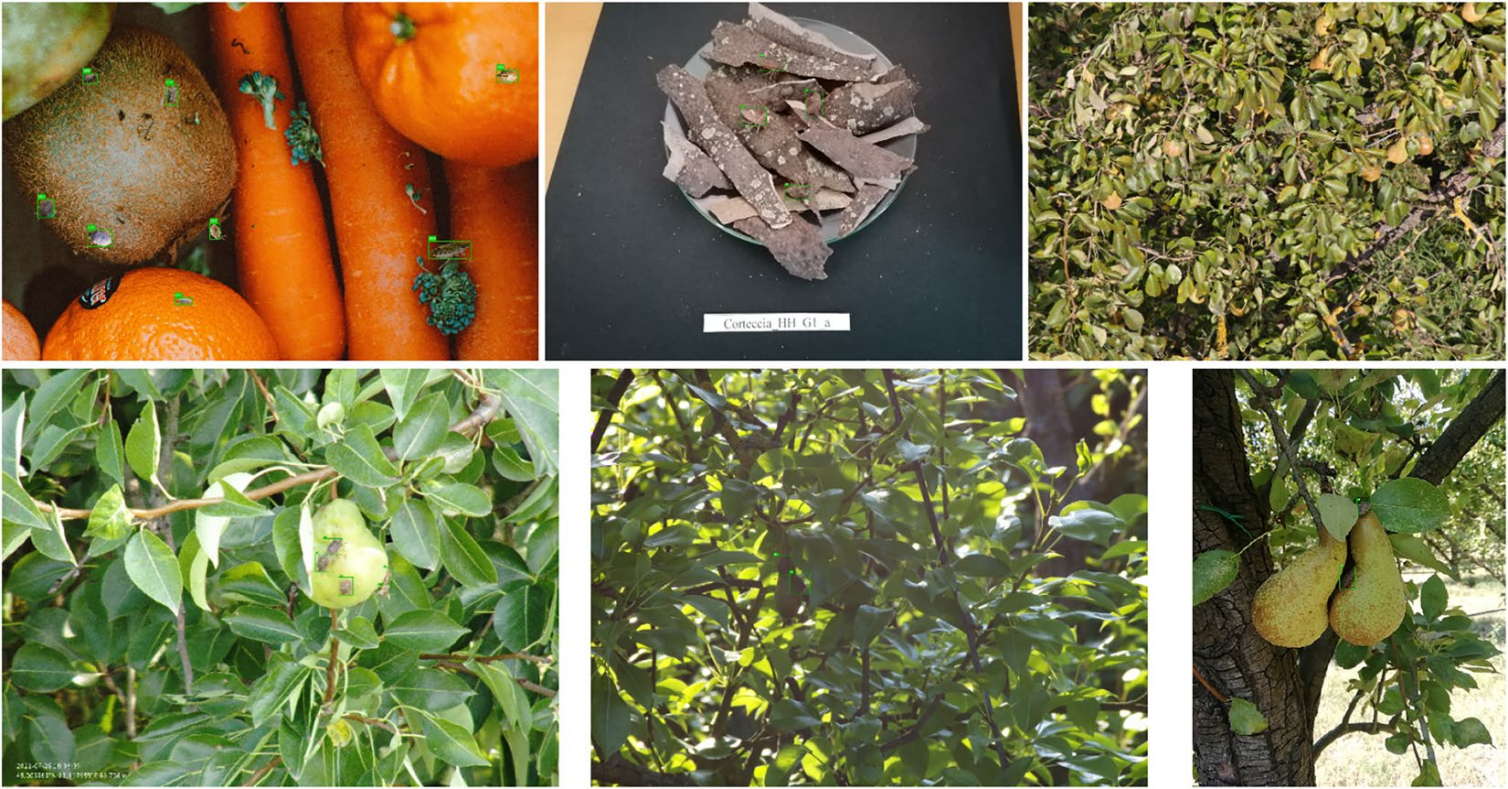
Representative example images from different acquisition modalities included in the dataset. HH are highlighted with green bounding boxes.

To illustrate the heterogeneity of the dataset, Fig. 6 shows representative example images from each acquisition modality, including UAV imagery at different viewing geometries, stationary-camera images, smartphone-acquired images, and synthetic images.

## Technical Validation

### UAV Image Annotation Quality

All UAV images included in the dataset were manually annotated and independently reviewed by two trained entomologists. Disagreements were discussed and resolved to obtain a consensus annotation set, ensuring consistent identification of small and partially concealed insects in complex canopy imagery. In cases of partial occlusion, bounding boxes encompass only the visible portion of the insect body; no attempt is made to estimate the full body extent beneath the occluding element (e.g., a leaf or branch).

### Stationary Camera Annotation Refinement

The full set of 2023 stationary-camera frames was re-examined using the improved monitoring workflow described in^16^. Automatic pre-screening was followed by systematic manual inspection. This procedure revealed additional HH individuals not captured during the initial pass, including insects partially occluded by leaves or present under low-contrast conditions. All newly detected individuals were incorporated into the released annotations. In cases of partial occlusion, bounding boxes encompass only the visible portion of the insect body; no attempt is made to estimate the full body extent beneath the occluding element (e.g., a leaf or branch).

### Environmental Data Quality Control

Environmental time series collected in 2023 underwent basic quality control prior to release. Timestamps were checked for consistency across sensor nodes. Missing data were left as explicit gaps without interpolation, providing a transparent record of sensor availability.

## Usage Notes

All object annotations are provided in YOLO format, where each annotation file contains one row per object with the following fields: class identifier, normalized horizontal and vertical coordinates of the bounding box center, and normalized bounding box width and height. Coordinates are expressed relative to the image dimensions and range between 0 and 1. Annotation files share the same base filename as their corresponding images.

The core modality archives include only images containing at least one annotated insect. In addition, the public release includes four dedicated empty-image archives (negative-uav-2021/2022/2023 and negative-stationary), provided as image-only support data without annotations. For stationary cameras, negatives were sampled uniformly at 10 images/day across 174 acquisition days (May–November 2023), for a total of 1,740 images. For UAV data, negatives were sampled across multiple acquisition days in each campaign year: 202 images (2021), 413 images (2022), and 413 images (2023). The corresponding UAV day-level coverage is 5 acquisition days in 2021, 21 in 2022, and 25 in 2023. For UAV data, each mission defines a time window from which negatives are drawn at random up to a fixed per-window cap, yielding a balanced spread of negatives across missions.

Users training detection or classification models with this dataset may consider the following practical recommendations.

Images span a wide range of spatial resolutions depending on the acquisition modality. UAV images have a resolution of 5184 × 3888 pixels, stationary-camera images 4056 × 3040, laboratory images 4000 × 3000, artificial images 2000 × 1500, and smartphone images exhibit device-dependent variability. Concerning both UAV images collected with Protocol 1 and the stationary camera set, resizing images to 640 × 640 pixels yields promising detection performance^11^. On the other hand, since Protocol 2 returns images where individuals appear significantly smaller than in other modalities, simple resizing leads to poor performance. To address this issue, images are partitioned into spatial tiles, which preserves the effective size of the insects and improves detection accuracy as shown in^17^. All images are provided in RGB color space.

We adopt a *regular sampling* strategy, in which each image is divided into non-overlapping patches of size 640 × 640 px using a regular grid. Since the original image dimensions are not necessarily divisible by 640, zero-padding is first applied to the right and bottom borders so that both width *W* and height *H* become multiples of 640. The padded image is then split into tiles, and the corresponding annotations are remapped to each patch. This procedure produces a total of $\lceil \frac{W}{640}\rceil \times \lceil \frac{H}{640}\rceil$ patches per image. For example, an image of size 5184 × 3388 pixels results in 7 × 6 tiles after padding. Further details on the impact of this strategy on detection performance are provided in^17^.

The dataset is highly imbalanced with respect to species representation, with the majority of annotated bounding boxes corresponding to HH, as illustrated in Table 3. Users may consider class-weighted losses, resampling strategies, or data augmentation techniques to mitigate this imbalance during training. Given the extreme class imbalance and temporal concentration of NZ (predominantly in 2021), the dataset should be considered primarily as an HH detection resource; NZ is provided as an auxiliary minority class rather than as a balanced target for multi-class benchmarking.

UAV imagery collected in 2021 differs from that collected in 2022 and 2023 in terms of flight altitude (see Fig. 2), while the same camera, crop type (pear orchards), and seasonal period were used across all years. Imagery from 2022 and 2023 was acquired using the same flight altitude. Users combining data across years may account for these differences when defining training and evaluation splits. For protocol-aware reuse, we recommend reporting results separately for Protocol 1 (2021) and Protocol 2 (2022–2023) before pooled analyses. When evaluating cross-domain robustness, users should include year-/protocol-disjoint settings (e.g., train on Protocol 2 and test on Protocol 1, and vice versa) and explicitly report transfer performance. Protocol-specific preprocessing can be considered; in particular, tiling may be beneficial for Protocol 2, where insects typically appear smaller. As the dataset is based on a single cultivar, between-cultivar variability is not represented; observed appearance variability is primarily intra-cultivar and driven by phenology, canopy structure, and environmental conditions over time.

Multiple insects are frequently present within the same image, often with two individuals appearing simultaneously. Bounding boxes may therefore overlap or be closely spaced, particularly in high-density scenes.

Users should be aware of several factors that may affect downstream analysis. Lighting conditions vary substantially across acquisition settings and times of day, resulting in hard shadows, reflections, and high-contrast regions in some images. In natural scenes, insects may be partially occluded by leaves or branches, and in some cases only a portion of the insect body is visible. These factors may influence detection performance and should be considered during model design and evaluation.

Environmental data from the stationary sensor nodes are provided as archived time-series records. During the summer period, environmental measurements are temporally aligned with stationary-camera acquisitions at the level of day and hour. These data are intended to support analyses that relate insect presence and activity to microclimatic conditions, such as temperature, humidity, light intensity, wind-related measurements, and leaf wetness^18,19^.

Environmental measurements were recorded at short intervals; however, due to sensor outages and transmission interruptions, some variables exhibit irregular sampling and discontinuous timestamps. Missing data are not gap-filled and are represented by absent records, allowing users to apply application-specific preprocessing, aggregation, or filtering strategies. Timestamps in the exported tabular weather data follow RFC3339 UTC format (suffix Z).

Users analyzing environmental data in conjunction with image-based observations are advised to explicitly handle temporal alignment, sampling irregularities, and missing intervals according to their application requirements.

All files are provided using open and widely supported formats, including JPEG and PNG for images, TXT for annotations, and archived InfluxDB v2.7.12 time-series files for environmental data. The dataset is intended to support reuse across a range of computer vision and ecological modeling workflows.

Users are encouraged to cite both the dataset and the associated publication when using these data in derived works. Relevant references describing the UAV acquisition protocol and sensor deployment are provided in the reference list.

## Data Availability

All data described in this article are publicly and openly available via the Zenodo repository^4^. The dataset concept is 10.5281/zenodo.18045392; the exact released version for this manuscript is 10.5281/zenodo.20431348. The repository contains all raw images, annotation files, and environmental time-series data in the directory structure described in the Data Records section.

## Footnotes

1 https://docs.influxdata.com/influxdb/v2/query-data/

## Code availability

All code used to generate the dataset summary statistics and figures presented in this article is publicly available at https://github.com/TheAnswer96/haly-data-summary.
